# CHRONIC DISEASE, DEPRESSION, AND SUICIDAL IDEATION IN OLDER ADULTHOOD: THE ROLE OF INTERPERSONAL FACTORS

**DOI:** 10.1093/geroni/igae098.3429

**Published:** 2024-12-31

**Authors:** Kallisse Dent, Linh Dang, Viktoryia Kalesnikava, Briana Mezuk, Leah Richmond-Rakerd

**Affiliations:** University of Michigan, Ann Arbor, Michigan, United States; University of Michigan, Ann Arbor, Michigan, United States; University of Michigan, Ann Arbor, Michigan, United States; University of Michigan, Ann Arbor, Michigan, United States; University of Michigan, Ann Arbor, Michigan, United States

## Abstract

Suicide risk increases in older adulthood, and new chronic-disease diagnoses can be a precipitating factor. Motivated by the interpersonal theory of suicide, we tested interpersonal constructs --thwarted-belongingness, perceived-burdensomeness, and hopelessness -- as mediators of associations of chronic-disease with depression and passive suicidal ideation (PSI). Data were from the Health and Retirement Study, a nationally-representative, longitudinal cohort of older adults. We identified incident chronic-disease diagnoses and changes in interpersonal factors over a four-year period (2010-2014 or 2012-2016). Depression and PSI were assessed two years later (2016 or 2018) using the Composite International Diagnostic Interview- Short Form. We measured thwarted-belongingness by social contact, positive and negative social support, loneliness, and community engagement; perceived-burdensomeness by activities of daily living (ADLs), instrumental ADLs, financial strain, number of helpers, and employment status; and hopelessness using a four-item scale. Statistical analyses accounted for complex survey design and adjusted for demographics, baseline health characteristics, and the outcome at baseline. Older adults with a newly diagnosed chronic-disease, relative to those without a new diagnosis, were at elevated risk for depression (odds ratio (OR)=1.34 [1.03-1.75]) and PSI (OR=1.63 [1.26-2.11]). They also experienced greater increases in thwarted-belongingness (β=0.11 [0.01-0.20]), perceived-burdensomeness (β=0.12 [0.06-0.15]), and hopelessness (β=0.07 [0.01-0.13]) following diagnosis. Accounting for these changes in interpersonal factors modestly attenuated associations of new chronic-disease diagnoses with depression and PSI. New chronic-disease diagnoses antedate depression and PSI, even within a population with pre-existing chronic-disease burden. Targeting interpersonal factors, including thwarted-belongingness, perceived-burdensomeness, and hopelessness, following incident chronic-disease diagnoses may modestly reduce depression and PSI risk.

